# Quantifying flight aptitude variation in wild *Anopheles gambiae* in order to identify long-distance migrants

**DOI:** 10.1186/s12936-020-03333-2

**Published:** 2020-07-22

**Authors:** Roy Faiman, Alpha S. Yaro, Moussa Diallo, Adama Dao, Samake Djibril, Zana L. Sanogo, Margery Sullivan, Asha Krishna, Benjamin J. Krajacich, Tovi Lehmann

**Affiliations:** 1 Laboratory of Malaria and Vector Research, National Institute of Allergies and Infectious Diseases, The National Institutes of Health, Rockville, MD USA; 2 Malaria Research and Training Center, Faculty of Medicine, Pharmacy and Odonto-Stomatology, Bamako, Mali

**Keywords:** *Anopheles gambiae*, Flight-aptitude, Migration, Seasonality, Sahel

## Abstract

**Background:**

In the West African Sahel, mosquito reproduction is halted during the 5–7 month-long dry season, due to the absence of surface waters required for larval development. However, recent studies have suggested that both *Anopheles gambiae* sensu stricto (*s.s*.) and *Anopheles arabiensis* repopulate this region via migration from distant locations where larval sites are perennial. *Anopheles coluzzii* engages in more regional migration, presumably within the Sahel, following shifting resources correlating with the ever-changing patterns of Sahelian rainfall. Understanding mosquito migration is key to controlling malaria—a disease that continues to claim more than 400,000 lives annually, especially those of African children. Using tethered flight data of wild mosquitoes, the distribution of flight parameters were evaluated as indicators of long-range migrants *versus* appetitive flyers, and the species specific seasonal differences and gonotrophic states compared between two flight activity modalities. Morphometrical differences were evaluated in the wings of mosquitoes exhibiting high flight activity (HFA) vs. low flight activity (LFA).

**Methods:**

A novel tethered-flight assay was used to characterize flight in the three primary malaria vectors- *An. arabiensis, An. coluzzii* and *An. gambiae s.s*. The flights of tethered wild mosquitoes were audio-recorded from 21:00 h to 05:00 h in the following morning and three flight aptitude indices were examined: total flight duration, longest flight bout, and the number of flight bouts during the assay.

**Results:**

The distributions of all flight indices were strongly skewed to the right, indicating that the population consisted of a majority of low-flight activity (LFA) mosquitoes and a minority of high-flight activity (HFA) mosquitoes. The median total flight was 586 s and the maximum value was 16,110 s (~ 4.5 h). In accordance with recent results, flight aptitude peaked in the wet season, and was higher in gravid females than in non-blood-fed females. Flight aptitude was also found to be higher in *An. coluzzii* compared to *An. arabiensis*, with intermediate values in *An. gambiae s.s*., but displaying no statistical difference. Evaluating differences in wing size and shape between LFA individuals and HFA ones, the wing size of HFA *An. coluzzii* was larger than that of LFAs during the wet season—its length was wider than predicted by allometry alone, indicating a change in wing shape. No statistically significant differences were found in the wing size/shape of *An. gambiae s.s.* or *An. arabiensis*.

**Conclusions:**

The partial agreement between the tethered flight results and recent results based on aerial sampling of these species suggest a degree of discrimination between appetitive flyers and long-distance migrants although identifying HFAs as long-distance migrants is not recommended without further investigation.

## Background

The long-distance migration (LDM) of insects [1–4] has provided primarily drawbacks to the economy, human agriculture, and health [5]. For example, LDM has had impacts on food security [1, 6–11], public health [12–14], and even the transfer of nutrients by migrating insects [15–17]. Here, migration is defined as the persistent movement of individuals not driven by immediate cues for food, reproduction, or shelter, and which has a probability to land the migrator in a new environment suitable for survival/breeding [2, 3, 18]. The primary focus of this work is with the Sahelian Zone of West Africa, where mass seasonal migrations of pest insects, such as grasshoppers and pyrrhocorid bugs, into- and back out of the Sahel in Mali and Niger have been described [19–22]. These migrations follow cyclical shifts in wind direction as the Inter-Tropical Convergence Zone (ITCZ) moves north during March–August, then south during September–February, with the migrants taking advantage of ephemeral, but seasonally available, dependable habitats. However, because it is easier to notice immigration into areas depleted of conspecific populations, many other cases of insect migration have likely been discounted.

Anecdotal evidence has suggested that mosquitoes could also engage in long-range wind-assisted dispersals [23–27]. The prevailing view has been that such movements are accidental in most disease vector species and thus are of negligible epidemiological significance [28, 29]. However, a recent aerial sampling study showed that in the Sahel, many species of mosquitoes, including *Anopheles coluzzii*, *Anopheles gambiae* sensu stricto (*s.s*.), and several secondary malaria vectors, regularly engage in seasonal flights 40–290 m above ground [30]. Because of the large number of migrants, most of which were gravid females, and the large distances they were able to cover, the likely epidemiological significance of these migrations are inescapable.

As a previously unrecognized behaviour in malaria mosquitoes, windborne long-distance migration raises many questions. These include: What fraction of the population migrate (i.e. are they partial migrators)? Are migrants more common in some species and under certain conditions, and if so, what might these be? What are the physiological and molecular mechanisms involved in preparing for and undertaking the journey? Addressing such questions using aerial sampling would be challenging for many insect species because they would be intercepted at a frequency of less than one per sampling night [30].

Tethered-flight mills have been used extensively to characterize short- and long-distance flyers in many insect species [31]. These include cotton strainers (*Dysdercus fasciatus*, Pyrrhocoridae) [32], corn leafhoppers (*Dalbulus maidis*, Cicadellidae) [33], the brown marmorated stinkbug (*Halyomorpha halys*, Pentatomidae) [34], and Buprestid beetles [35], facilitating investigations to address questions such as those mentioned previously. However, despite their intuitive appeal, flight mill results have also been reported to be at odds with expectations in species with well-established migration [36–38]. Thus, the approach has its merits and drawbacks [31, 35, 39] and predicting when it would be useful is not always clear. Additionally, while flight mills might be well suited for laboratory studies, they can be challenging in field experiments. Here, a novel assay was developed to measure the flight of tethered mosquitoes under field conditions using a fixed tether (non-rotary) and sound recordings to monitor flight. As previously done with flight mills, flight aptitude measures, such as total time in flight during the assay, may help distinguish persistent flight. Persistent flight, in turn, is presumably enhanced in migrants which fly considerably longer distances (= duration) than ‘appetitive’ flyers. Therefore, the aim was to estimate the fraction of strong flyers (presumed migrants) among wild mosquitoes, representing different species during different seasons. Based on population dynamics results [40–42], we initially predicted a high fraction of migrants during the early and late rainy season, along with a lower fraction of migrants in *An. coluzzii* (present in the Sahel during the dry season) compared to *An. gambiae s.s.* and *Anopheles arabiensis* (which are absent during the dry season).

Recent aerial sampling data [30], which more directly reflect flight activity prompted reformulation of the predictions. Instead of predicting migrants to peak in *An. gambiae* and *An. arabiensis* in the early wet and dry seasons species, the predictions now included: (a) migration would be seen across the three species, peaking in the mid and late wet season, and (b) gravid females will exhibit greater flight than unfed mosquitoes. Finally, a morphological investigation was added to assess whether putative migrants (based on flight data) exhibited a different wing morphology (i.e., size or shape), aiming to identify external features which can help in quantifying of potential migrators in a population.

## Methods

### Study area

Tethered-flight assays were conducted in the Sahelian village of Thierola (−7.2147 E, 13.6586 N) from August 21, 2015 until November 21, 2015 and from March 28 until September 27, 2016. During the dry season, due to a scarcity of mosquitoes in the Thierola area, assays were conducted in the rice-cultivation town of Kangaré (Selingue commune, −8.198 E, 11.644 N, 250 km SSW of Thierola) between December 24, 2015 and February 12, 2016.

### In total, 114 assay-nights were conducted in the field throughout the course of 13 months during the rainy season (June–October) and during the dry season (November–May).

In both villages, flight assays were conducted indoors, within local houses that were selected for experimentation. Windows in the experimental rooms enabled limited natural light without measurable wind or air currents. In Thierola, the mean nightly (21:00 to 5:00 h) temperature throughout the rainy season was 24.5 °C (range: 20.2–32.4 °C), with a mean RH of 88.6% (range: 31–100%). During the dry season, the mean nightly temperature was 23.4 °C (range: 10.9–35.6 °C) with a mean RH of 27.3% (range: 5.0–100%). In Kangaré, the mean nightly temperature between December and February was 26.2 °C (range: 24.5–27.7 °C) with a mean RH of 29.3% (range: 20.9–63.1%).

### Mosquitoes

Wild *An. gambiae* sensu lato (*s.l.)* were collected within the village, both indoors and outdoors, on the morning of a flight assay (between 07:00 and10:00 h). They were collected using aspirators and kept indoors in 1-gallon plastic cages covered with a dampened cloth. Mosquitoes were then provided water on cotton balls for hydration until 16:00 h. Before each flight assay, active mosquitoes (reacting with flight to tapping on the cage) were selected by gonotrophic stage (gravid or unfed), which was assessed by visual examination of the abdomen.

Following morphological identification [43], only *An. gambiae s.l.* were included in the flight assays. Subsequent species identification was performed by species-specific PCR and PCR–RFLP using legs as a template [44]. Thirteen individuals not identified by this assay were excluded.

### Wing measurements

**Fig. 1:**
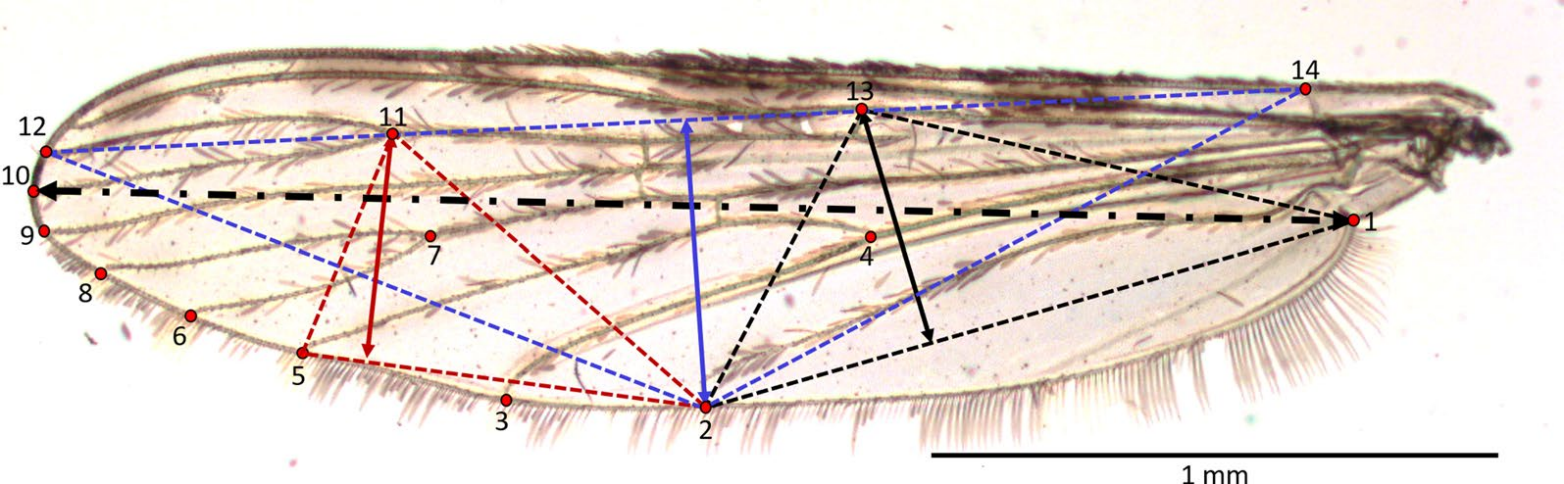
Wing of *Anopheles gambiae s.l.* (×25 magnification); Landmarks (14) denoted by numbers. Black dot-dash line represents the wing length between landmarks 1–10. Wing width was the average value of the height of three triangles formed between landmarks: 1-2-13 (proximal triangle, black), 2-5-11 (distal triangle, red), and 2-12-14 (medial triangle, blue). Scale bar = 1 mm

Wing length (WL) and wing width were measured as described elsewhere [45]. Wings were spread under a coverslip with glycerol and photographed at ×25 magnification using a microscope (Olympus DM-4500B) coupled with digital camera (MC170 HD, Leica Microsystems, Wetzlar, Germany). For each wing, 14 specific landmarks (Fig. 1, i.e., vein intersections) were mapped using the tps-DIG32 2.15 software package [46]. Wing length was measured between points 1 and 10 (Fig. 1) and wing width was calculated as the average value of the height of the three triangles formed between the landmarks (Fig. 1): 1-2-13 (proximal triangle), 2-5-11 (medial triangle), and 2-12-14 (distal triangle). For damaged wings (n = 265), wing length was predicted using a regression analysis based on distances between landmarks 1 and 7 for wings with a damaged tip or based on distances between landmarks 4 and 10 for wings with a damaged base. Regression models showed that these predictors accounted for > 95% of the variation in wing length based on intact wings.

### Tethered flight assay

**Fig. 2:**
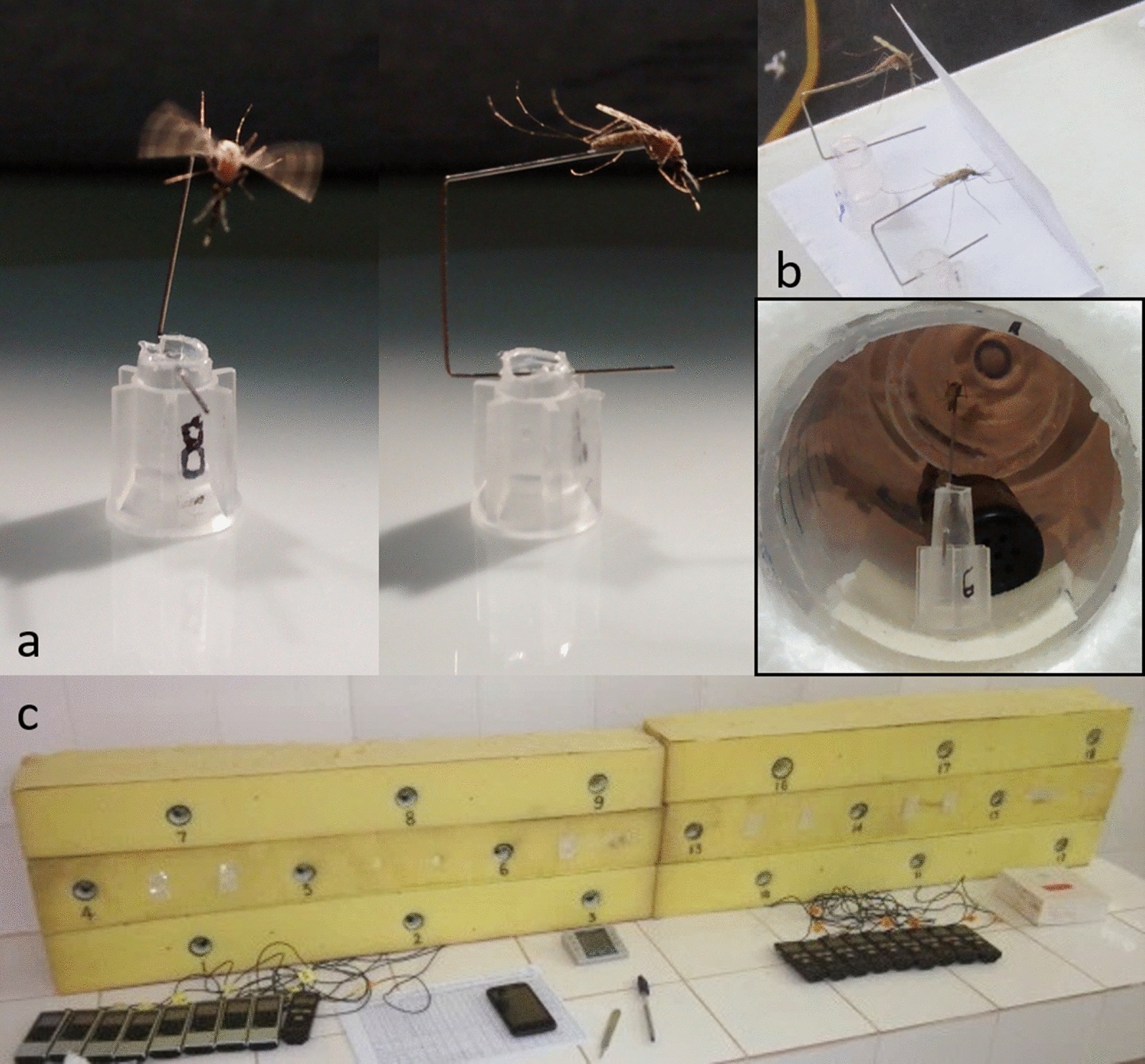
Tethered female *Anopheles gambiae s.l.***a** Entomological pin attached to ventral side of posterior abdomen of the mosquito (**a**; right), allowing unobstructed flight (**a**; left). Bottom part of pin inserted into clipped 10 µl pipet tip as a base. **b** Tethered mosquitoes in recuperation time before assay start, fore legs resting on folded paper for tarsal contact preventing flight. **c** Tether flight hive of 18 flight tubes housed inside soft (mattress) foam for surrounding sound muffling and external cue reduction. Each flight tube microphone connects to an individual sound recorder. **c**_i_ Tethered female inside flight tube (polystyrene prototype, not used in the experiment); tethered mosquito construct attached on to double-sided foam tape with microphone (black) in backdrop. Photos by: RF (**a** and **c**_i_) and ASY (**b** and **c**)

Individual mosquitoes were gently aspirated from their cages and transferred into a 1.6 mL microcentrifuge tube with the bottom removed and replaced with muslin netting. These tubes were then inserted into a 50 mL Falcon tube containing a cotton ball with 2–3 drops of diethyl ether (Cat. No. 673811, Sigma-Aldrich, St. Louis, MO) at the bottom. Mosquitoes were anesthetized by exposure to the ether-vapor rich environment for 3–4 s, then swiftly placed, wings down, under a dissection stereo-microscope (Zeiss Stemi 2000-C. Carl Zeiss Microscopy, Germany). Entomological pins (Morpho No.3. Ento Sphinx, Czech Republic), with the sharp ends clipped off, were then bent twice at 90° to result in a square bracket shape. The tip-end of the pin was lightly dipped in glue (Elmer’s, Glue-All E1322, Atlanta, GA) and gently pressed on to the ventral side of the posterior abdomen (covering the posterior half of the abdomen). Meanwhile, the other end was threaded through the base of a disposable 10 μl pipette tip with the nozzle cone (dispensing end) clipped off (Fig. 2a). Tether pins were cut to size and bent, enabling all the mosquito legs to remain suspended in the air throughout the assay. This, in turn, prevented tarsal contact and flight cessation. Tethered mosquitoes were allowed 2 h to fully recuperate from the anesthesia before the flight assay, during which time their fore legs were allowed to rest on a folded piece of paper (‘leg-rest’). This ‘leg-rest’ provided tarsal contact and prevented flight before the assay (Fig. 2b).

At the end of the recuperation time, tethered mosquitoes were inserted into individual flight tubes (50 mL Falcon^®^, Corning, NY, USA) housed within a polyurethane foam hive (foam mattress) for soundproofing and environmental cue reduction (Fig. 2c). Tether constructs (mosquito, pin and base; Fig. 2a) were secured onto a small piece of double-coated urethane foam tape (Cat. No. 4026. 3 M^®^, St. Paul, MN) to fasten them at 1 cm inward of the flight tube edge. Each flight tube housed a small microphone (ME-15, Olympus America Inc., Center Valley, PA, USA) attached to a portable voice recorder (VN-5200PC, Olympus America Inc., Center Valley, PA, USA) (Fig. 2c and c_i_) to record flight sound.

### Flight sound extraction

Tethered mosquitoes were recorded over a 10-h period starting at 21:00 h; sound recordings were then downloaded and read using Audacity 2.1.2 open-source software [47]. Flight bouts (episodes of flight) were identified visually in spectrogram view (Additional file 1: Fig. S1a) and uncertain flights were confirmed by listening to the flight sound recordings. For each flight bout, start time and duration were manually logged into a Microsoft Excel^®^ spreadsheet. Since the shortest time frames measured by the software were 1-s long, all flight bouts shorter than 1 s were inserted into the database as 1 s long bouts. In total, 216 individual mosquito recordings from 47 different assay-nights were extracted manually. Subsequently, Raven Pro 1.5 Interactive Sound Analysis Software [48] was used to detect and extract flight bouts from the sound files. This software, by utilizing a Band Limited Energy Detector (BLED), estimates the background noise of a signal and uses this information to find sections of the signal exceeding a user-specified signal-to-noise ratio (SNR) threshold in a specific frequency band during a specified time [49]. BLED outputs were verified audibly or visually in spectrogram view to rule out false positive flight bouts (Additional file 1: Fig. S1b).

All 8-h long sound files (approximately 140 megabytes each) were split into four sections before analysis in Raven Pro 1.5. This allowed for modification of the sound detector (BLED) and thus adjustments for changing background noises throughout the night (e.g., filtering out background noise produced by passing vehicles, electricity generators, crickets, farm animals, rain, etc.), as well as to ensured sufficient computer processor memory for the BLED runs. Although the Raven Pro software detectors picked up flight bout durations as short as 0.01 s, flight bouts separated by rest periods < 1.45 s (Audacity counted values above 1.5 s as 2 s long) (12% of samples) were pooled as continuous flight bouts to ensure consistency with the manual extraction method (see above). The resulting flight duration values were essentially identical to the original values in total, mean, and longest flight, while also consistent with the manual data with respect to flight bouts.

Flight bout records produced by Raven Pro were exported as text files (.txt), which were then read into a singular sound database (including manual flight extraction files) using R-Studio (The datasets used and/or analysed during the current study are available from the corresponding author on reasonable request).

### Data processing and analysis

The data was trimmed to the interval of 21:00 h to 05:00 h, in an effort to avoid shorter recordings due to battery failure on some of the nights. However, flight bouts beginning before 05:00 h and continuing after this time were included in full.

To characterize flight aptitude of individual mosquitoes, their total-flight duration between 21:00 h and 05:00 h (sum of all flight bouts per mosquito), longest flight-bout duration, and the total number of flight bouts per mosquito were computed. Assuming that long-distance flyers would exhibit much higher values in at least one of these flight measures, when compared to the majority of the population, median-based robust statistics were used; a method often used to detect outliers based on the distance of a value in units of the median absolute deviation (MAD). Unlike the mean and the standard deviation, the median and MAD are not sensitive to extreme outliers; as a result, they are considered “robust” and better represent the population without being excessively skewed by extreme values. For these reasons, they are often used to detect outliers [50–53]. Following conventional guidelines, the threshold for outlier detection was set as follows: Values > 3.5 MAD units from the median were considered as “High Flight Activity” (HFA) and values < 3.5 MAD units from the median were considered as “Low Flight Activity” (LFA), or short-range flight. Unless a population is observed to be “on the move”, as are migratory swarms of locusts [54] or monarch butterflies [55], it was assumed that the fraction of individuals expressing migratory behaviour at any given moment was small [32–35, 56, 57]. This assumption is based on previous Mark-Release-Recapture (MRR) studies, which suggested that a sizeable proportion of the population remain near the area where they were marked; moreover, these studies suggest that the duration of the migratory phase lasts only a few days and is typically shorter than the non-migratory phase, which can last several weeks or months [58, 59]. These findings underscore the reasons behind identifying mosquitoes with extremely high flight aptitude values as putative long-distance migrants.

## Results

A total of 707 individual mosquito recordings (including the manual extractions) from 114 assays were included in the analysis (Table 1). Individual flights lasting longer than 10 min were recorded in 46 mosquitoes, while flights exceeding 30 min were recorded in 12 mosquitoes. Total flight ranged 1–16,107 s with a mean total of 1257.2 s.

**Table 1:** Mosquito samples by season, gonotrophic state and species, for which flight data was collected and analysed

Seasons	Gonotrophic stage	*An. coluzzii*	*An. arabiensis*	*An. gambiae*	Total
Dec–Feb	Gravid	62	0	2	64
	Unfed	2	0	0	2
Mar–Apr	Gravid	31	0	0	31
	Unfed	0	0	0	0
Jul	Gravid	75	0	3	78
	Unfed	0	0	0	0
Aug–Sep	Gravid	125	10	62	197
	Unfed	30	1	8	39
Oct–Nov	Gravid	114	95	76	285
	Unfed	5	5	1	11
Total		444	111	152	707

### Nightly flight activity and identification of putative long-distance flyers (LDMs)

**Fig. 3:**
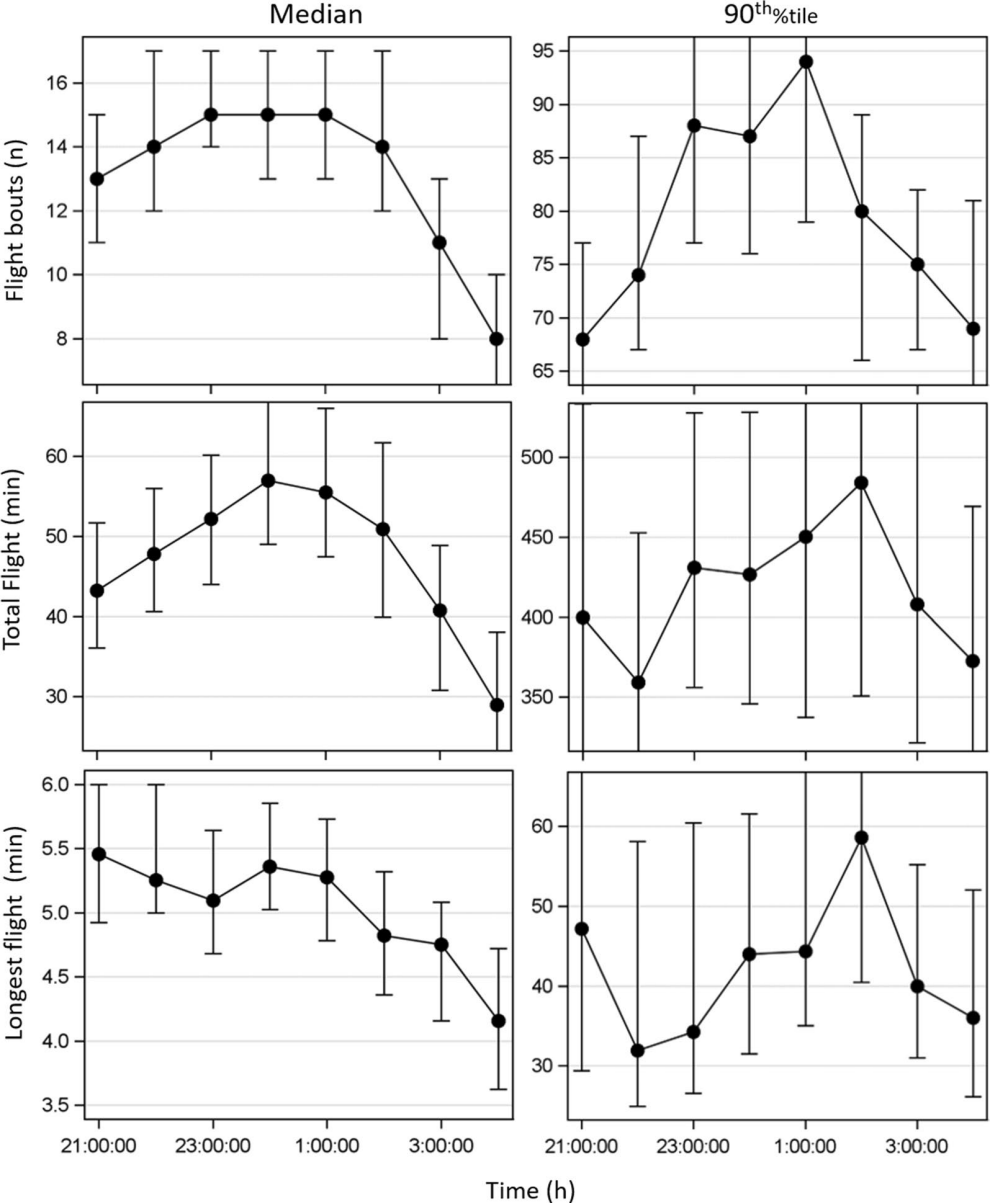
Nocturnal flight activity. Hourly flight bouts and total hourly flight across all 707 mosquitoes, (across species, season, and gonotrophic state). Hourly flight activity (flight aptitude) through the night showing the median (left column) and the 90th  %il (right column), representing trends of most mosquitoes and higher flight activity mosquitoes respectively. The 95% confidence interval of each hour (based on bootstrapping) not shown in full to emphasize the nightly trend

To determine if flight activity was concentrated in certain parts of the night, we examined three indices, namely (1) number of hourly flight bouts, (2) longest flight bout, and (3) total hourly flight (across species, season and gonotrophic state) (Fig. 3). To consider possible differences in nightly activity between appetitive and strong flyers, we evaluated both the median and 90th percentile of each flight aptitude index.

Overall, there were no significant peaks of activity identified in the hourly flight data. Variation, as measured by hourly 95% confidence intervals, was also found to be non-indicative of clear modality. Although there was a mild modality, suggesting elevated total flight and flight bouts between 11:00 and 02:00 h (but not in longest flight), this modality was not found to be statistically supported by the 95% CI which overlapped widely. It was concluded that the flight activity was spread homogenously throughout the assay time and used the full length/duration (21:00–05:00 h) to measure flight aptitude.

### Identification of putative migrants

**Fig. 4:**
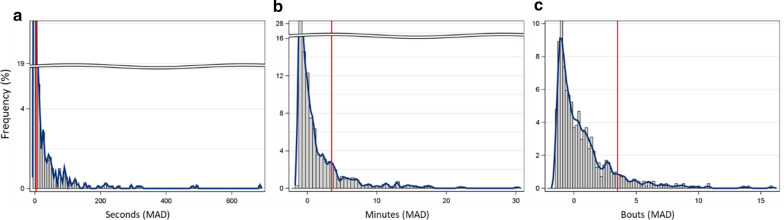
Flight aptitude index distributions: longest flight (**a**), total flight (**b**), and flight bouts (**c**). Flights were divided into two classes (x-axis; MAD units): LFAs; below 3.5 (left of vertical red line), and HFAs; above 3.5 (right of vertical red line) based on guidelines for outlier detection (see “Methods” section). The data are based on 707 wild female mosquitoes representing three species, both unfed and gravid females. Y-axis denotes the frequency in percent of the sample

Considering all recorded mosquito flights (n = 707 mosquitoes), the asymmetric distributions of each flight aptitude index revealed a long right tail. This observed distribution corresponds to existing literature, with frequency distributions of laboratory-measured bouts of flight [60, 61] showing a majority of individuals making short flights and a only few making long ones (Fig. 4).

Following previous flight behaviour studies [31–34, 37, 62], the mosquitoes at the far-right tail of the distribution were suspected to represent long-distance flyers, or HFA individuals, in our study. Subsequently, differences in the proportion of HFAs among species, seasons, and gonotrophic state were evaluated.

For the most part, flight aptitude indices were significantly correlated with each other; however, correlation coefficients (Spearman) were negative (r = −0.43) between the longest flight bout and the number of flight bouts and moderately positive (r = 0.32–0.52) between total flight and other indices (Additional file 1: Fig. S2). The relatively low correlation coefficients indicate that each flight index conveys unique information—the negative correlation suggests that flight bouts describes “restlessness” unlike “flight persistence” that is captured by the longest flight bout and especially total flight. Therefore, all three indices were compared to determine which were more important/informative predictors of migrants.

### Variation of flight aptitude by season

**Fig. 5:**
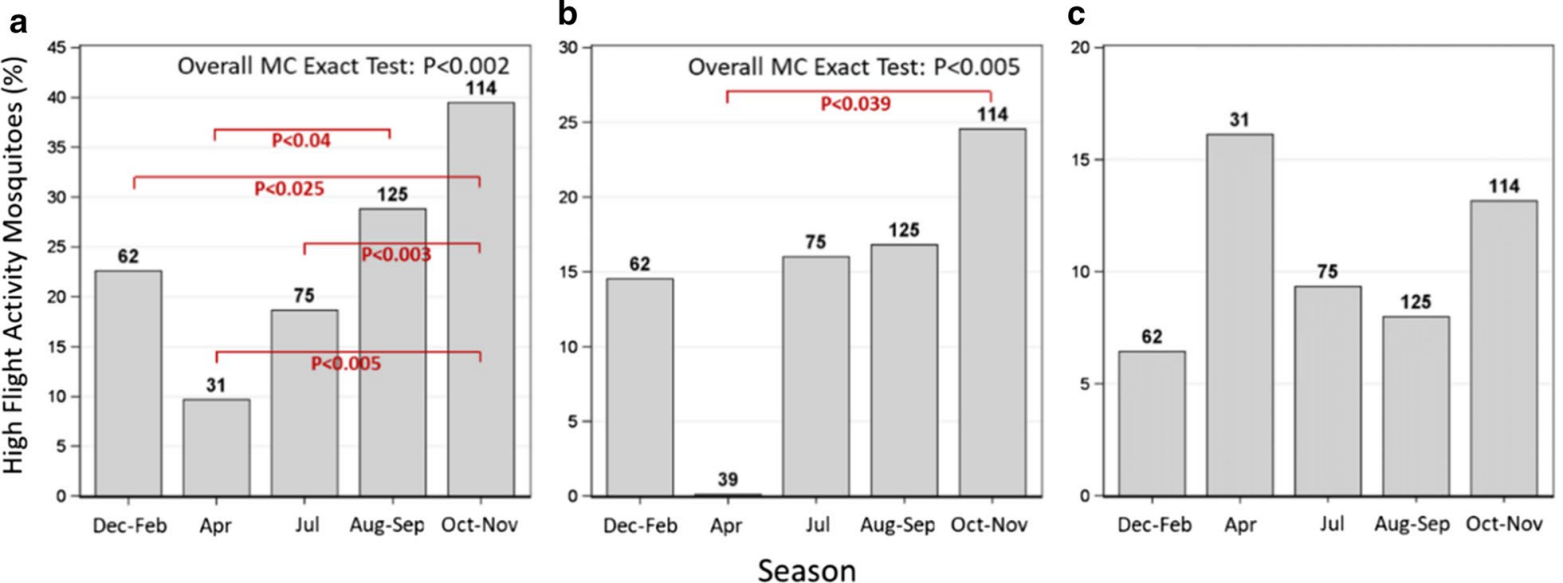
Variation of flight aptitude indices by season; longest flight (**a**), total flight (**b**), and flight bouts (**c**). Variation in *Anopheles coluzzii* flight aptitude between seasons; The x-axis depicts the different parts of the year (‘seasons’); Dec–Feb and Apr represent the dry season. Jul, Aug–Sep and Oct–Nov represent the wet season. Y-axis values are percent frequencies of the HFA populations, with *n* above each bar. Seasonal flight aptitude comparison was carried out on gravid *An. coluzzii* females, the only species which had samples across seasons

Seasonal variation in flight aptitude was tested on gravid *An. coluzzii*, as this was the only species (and gonotrophic state) found across seasons. Significant differences were most pronounced when looking at longest flight bout (P < 0.002, overall Monte-Carlo Exact test), but were also detected when examining total flight (P < 0.005, overall Monte-Carlo Exact test) (Fig. 5a and b, respectively). With regards to the longest flight bout index, the highest fraction of HFA was discovered in the late wet season (Oct–Nov; 39.5%), followed by the mid-wet season (Aug–Sep; 29%), early wet season (Jul; 19%), early dry season (Dec–Feb; 22%), and finally, the lowest fraction being in the late dry season (Apr; 10%, P < 0.005, 2-tailed test, Fig. 5a). A similar trend, albeit with smaller differences, was detected when looking at total flight, with only a single significant difference between late wet season and late dry season (P < 0.04, Fig. 5b). Flight bouts did not follow this pattern and showed no significant difference in the overall test (Fig. 5c).

### Variation of FA between species

**Fig. 6:**
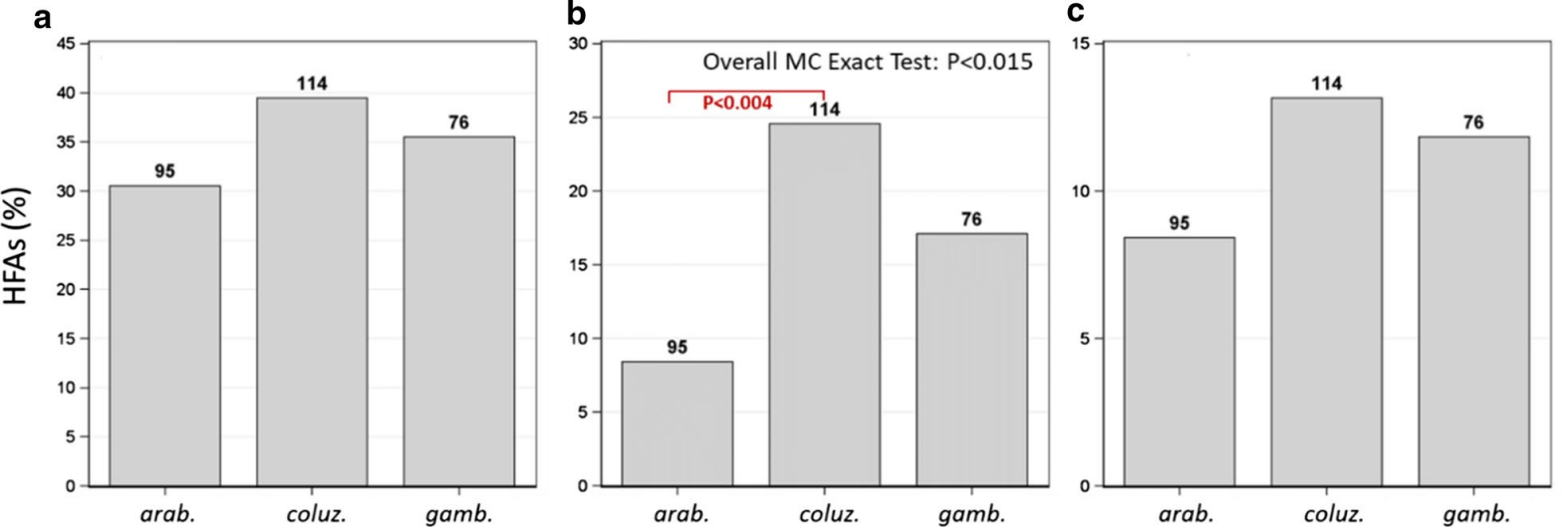
Variation of flight aptitude between species (gravid females in Oct–Nov, when sample sizes were sufficient); longest flight (**a**), total flight (**b**), and flight bouts (**c**). Values are percent frequencies of the flyer populations, with *n* above each bar. Overall test is a contingency table exact test using Monte Carlo with 10,000 replicates (P-values pertain to 2-sided tests). Specific comparisons are shown were tested using contrasts in logistic regression if overall test was significant

Variations in flight aptitude between species were tested on gravid females between Oct and Nov when all species were represented. Considering total flight, *An. coluzzii* exhibited a significantly higher fraction of HFAs (25%) than *An. arabiensis* (8%), with *An. gambiae* displaying intermediate values (17%) (Overall Monte Carlo Exact Test χ^2^ = 10.0, P < 0.015) (Fig. 6b). Contrasting tests between species showed a significant difference between *An. coluzzii* and *An. arabiensis* (Wald χ^2^ = 8.7, P < 0.004). Although not statistically significant, similar trends were revealed in both longest flight and flight bout indices (Fig. 6a and c).

### Variation in flight aptitude between gonotrophic stages

**Fig. 7:**
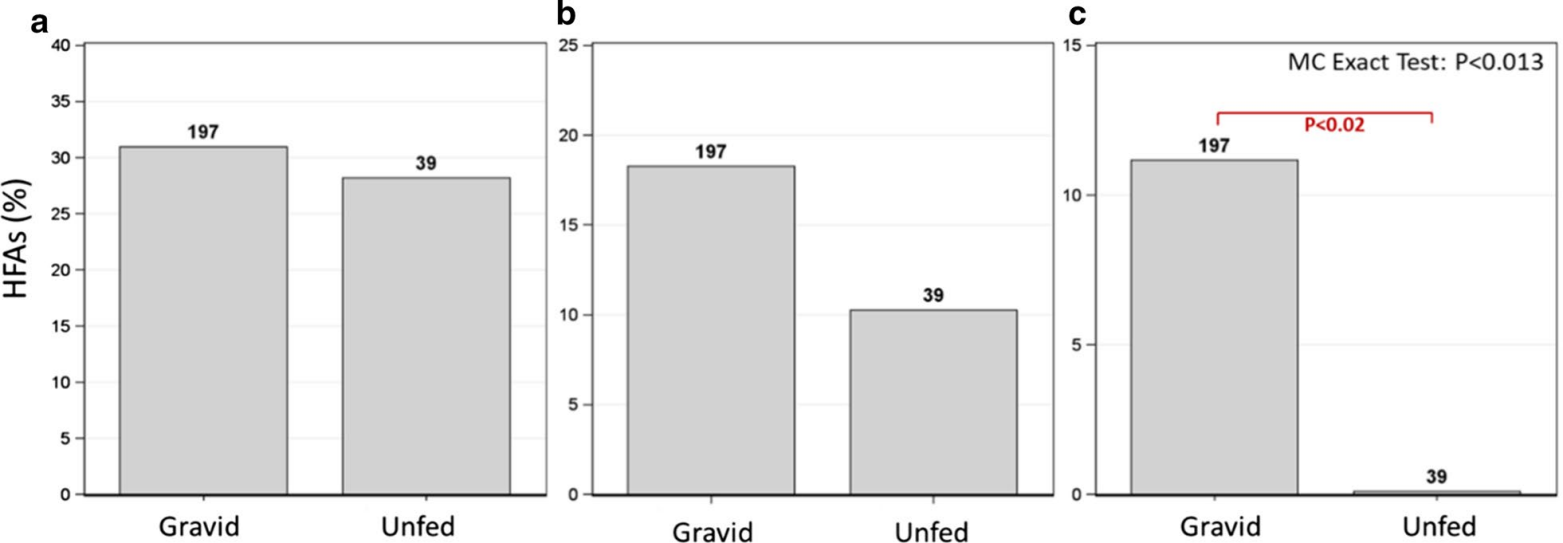
Variation in flight aptitude between gonotrophic stages; longest flight (**a**), total flight (**b**), and flight bouts (**c**). Overall test is a contingency table exact test using Monte Carlo simulations with 10,000 replicates. This Gonotrophic state comparison was done when on pooled species in Aug–Sep, when sample size was suitable for comparison. Prior CMH test showed that the effect was significant across species (not shown) and no heterogeneity between species was detected

The effect of gonotrophic stages on flight aptitude was tested after pooling the species, as well as with stratification by species (CMH test) in August–September, when the number of unfed females was suitable for such a test. These tests revealed that a significantly higher rate of HFAs among gravid females (11.2% vs. 0%, P < 0.013 Overall Monte Carlo Exact Test) was detected in flight bouts (pooled; P < 0.024, 1-tailed Fisher Exact test, and when stratified (CMH = P < 0.049, 2-tailed test) (Fig. 7c). However, while there were no significant differences detected when looking at the longest- and total flight indices, a consistent trend of higher HFA among gravid females was observed (Fig. 7a and b, respectively).

### Wing morphology and flight aptitude

**Fig. 8:**
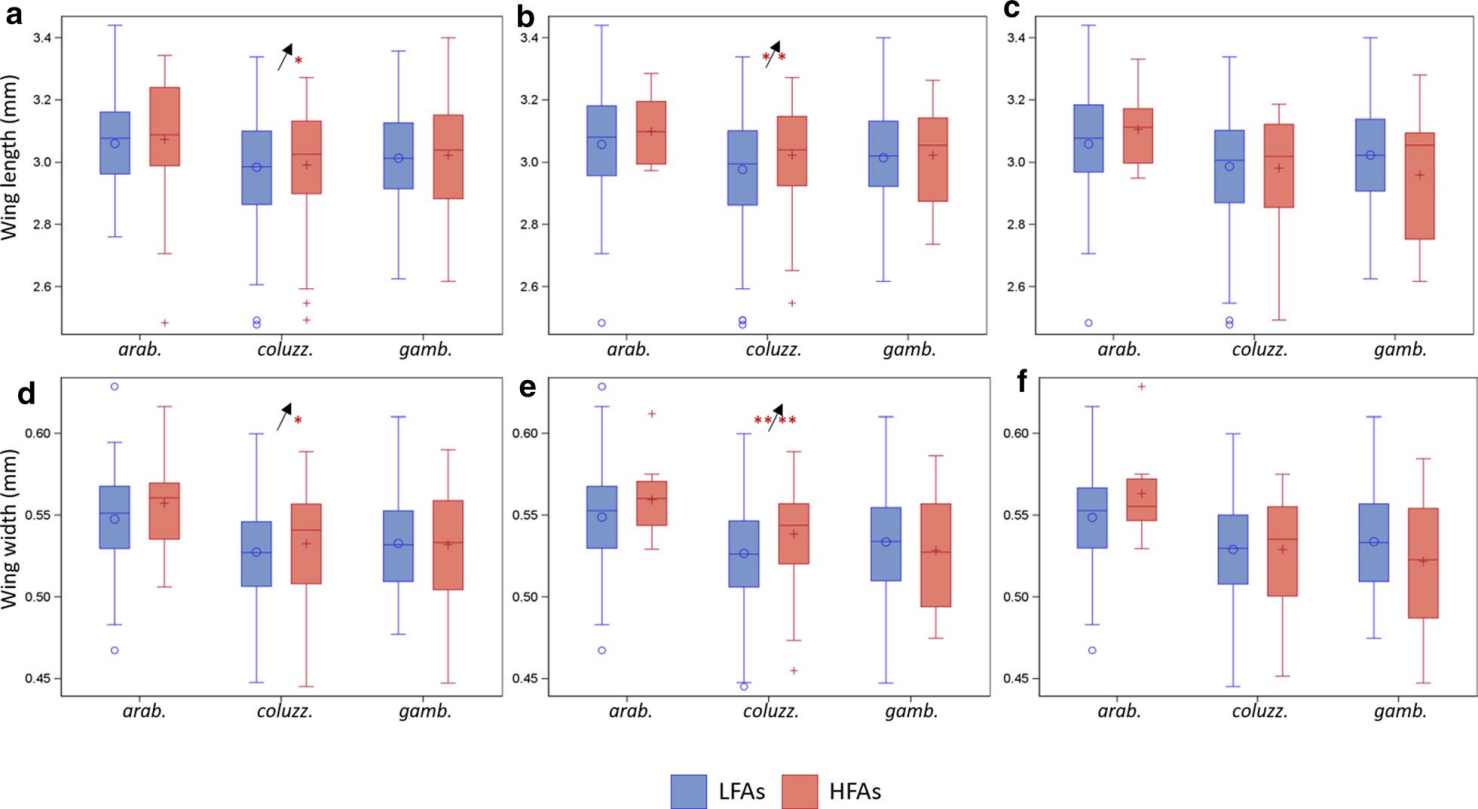
Box-whisker plot of wing length (top), and width (bottom) across species (x-axis, abbreviated species names: *arab. *= *An. arabiensis, coluz. *= *An. coluzzii., gamb. *= *An. gambiae s.s.*) in longest flight (**a** and **d**), total flight (**b** and **c**) and flight bouts (panels c and f) for LFAs (blue) and HFAs (red). Mean marked as ○ (for LFAs) or + (for HFAs). Horizontal line within box represents the median. Box bottom and top are 25th and 75th percentile, respectively, whiskers extend up to 1.5 time the inter-quartile range and outliers (‘o’ or ‘+’) represent observations that extend beyond the whiskers. Significantly larger mean and median wing dimensions in HFAs vs. LFAs indicated by asterisks on the left and right of the arrow (showing direction of increase; LFA or HFA), respectively. One tailed significance levels of P < 0.05 and P < 0.01 measured by ANOVA (left of arrow) or Median score tests (right of arrow); shown as ‘*’ and ‘**’ respectively

Among the three species, significant differences in flight activity were found in *An. coluzzii* with regard to longest flight duration (Fig. 8a and d) and total flight (Fig. 8b and e), showing both longer (Fig. 8a and b) and wider wings (Fig. 8d and e) in HFAs (red) (Overall ANOVA; P < 0.02).

### Allometry of wings to detect wing shape variation within HFA’s

**Fig. 9:**
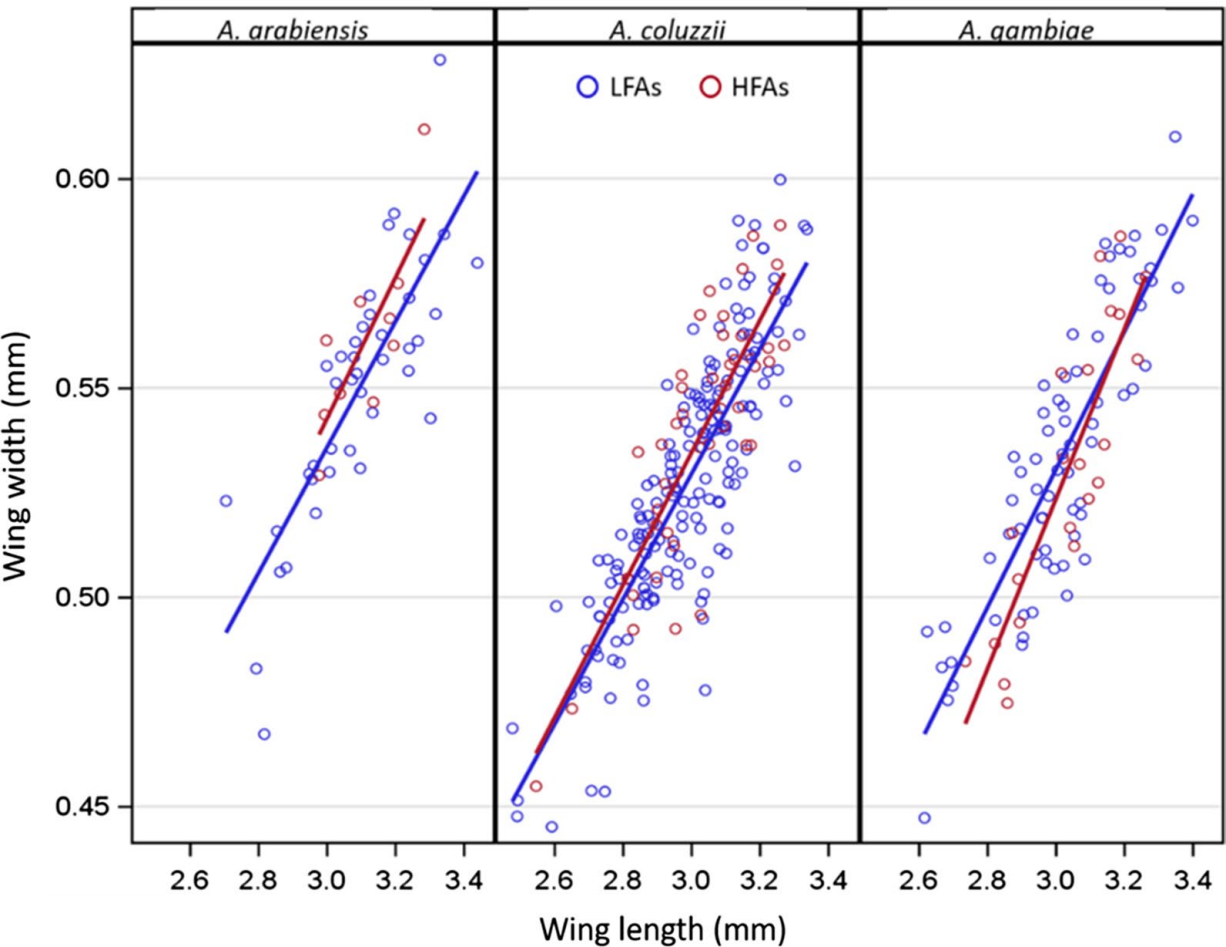
Wing allometry in HFAs (red) and LFAs (blue) for total flight across species (the lines shown were computed based on separate linear regression models for flyer type of each species). In gravid *Anopheles coluzzii* during the wet season, total-flight HFAs had wider wings than LFAs after adjusting for wing length (P < 0.035, 1-tail test)

In gravid *An. coluzzii* during the wet season, total-flight HFAs had wider wings when compared to LFAs, after adjusting for wing length (P < 0.035, 1-tail test) (Fig. 9). No significant interaction was found between wing length and HFAs, indicating that the shape effect was monotonic with wing length. A similar trend was observed in *An. arabiensis*, but no significant difference in intercepts was detected, possibly due to smaller sample size.

Consistent with previous studies [63, 64], *An. coluzzii* also displayed longer wings in the early dry season (December–February), when compared to other months in the year (Additional file 1: Fig. S3). This increase in length was isometric—in other words, it was accompanied by a proportional increase in wing width during this time (i.e. no change in wing shape).

## Discussion

Recent studies on windborne migration of African malaria vectors raise new questions about this previously unrecognized behaviour, including questions regarding the fraction of windborne migrants in the population, as well as how this fraction might change across species, seasons, or ecological zones, among other factors. Identification of migrants in field experiments could help address such questions. In this study, wild mosquitoes were subjected to a novel, field-adapted tethered-flight assay, in order to separate them into mosquitoes with high flight activity (HFA) or low flight activity (LFA), employing flight aptitude indices reflecting flight persistence (i.e., longest flight duration, and total flight) and restlessness (i.e., flight bouts). Albeit not without exceptions, based on previous flight-mill studies, HFA is likely to be more common in long-distance migrants than LFA [31, 37, 65, 66]. Accordingly, we evaluated variation in HFA mosquitoes, as putative migrants in Sahelian populations of *An. coluzzii, An. gambiae s.s*., and *An. arabiensis,* over various seasons and gonotrophic states. Although differences between groups were moderate, consistent with our predictions, we found elevated HFA mosquitoes in the wet season and among gravid females. However, predictions regarding species variation during the wet season were less certain. Based on Dao et al. [41], migrants were initially predicted only in *An. gambiae s.s*. and *An. arabiensis*; however, based on the aerial sampling of Huestis et al. [30], the presence of HFAs across all three species was predicted, with higher HFAs in *An. coluzzii*, followed by *An. gambiae s.s.* Consistent with Huestis et al. [30], results showed the highest proportion of HFA in *An. coluzzii*, with the lowest being in in *An. arabiensis*. Moreover, during the wet-season, *An. coluzzii* HFAs exhibited larger wings than conspecific LFAs.

Additional analysis indicated that wings of wet-season, *An. coluzzii* HFAs exhibited allometric change. Overall, these results agree with recent literature, which has found the dominance of gravid *An. coluzzii* flying during the wet season at altitudes of 40–290 m above ground [30].

Although an ultimate ‘comprehensive flight index,’ as well as cutoff values to distinguish between long-distance migrants and appetitive flyers are yet to be found, ad hoc indices and values have been successfully used, e.g., [33, 34, 36]. This study followed flight-mill based studies seeking to identify long-distance migrant insects that often relied on (1) total flight; (2) longest flight; and (3) the number of flight bouts [31, 37–39]. In this study’s findings, the low absolute value of the correlation coefficient between the longest flight bout and flight bouts (r = −0.43, Additional file 1: Fig. S3, bottom-right panel) highlights both high degree of independence of these indices and a degree of distinction between exhibiting flight persistence vs. restlessness. Only 10.5% of HFA mosquitoes based on longest flight were classified as such by flight bouts (unlike longest and total flight sharing 90.3% of HFAs), reaffirming that these modalities of flights are distinct. Overall, out of six comparisons (Additional file 1: Table S1), HFA mosquito comparisons based on total flight revealed significant differences in five tests, whereas the same comparisons based on longest flight and flight bouts revealed significant differences in three tests and one test, respectively. This suggests that persistence of flight is a more relevant modality for long-distance migration, similar to most other studies [37]. Notably, the variable longest flight bout showed consistent trends with total flight in five (of six) comparisons, whereas the variable flight bouts showed consistent trends in only two comparisons (Additional file 1: Table S1).

### Flight aptitude variation over seasons, species and gonotrophic states

Previous work in the Sahel has shown that *An. coluzzii* populations build up rapidly after the first rains (i.e. May–June), and decline towards the late wet season (i.e., October), presumably entering aestivation [41]. In contrast, both *An. gambiae s.s*. and *An. arabiensis* populations absent during the dry season build up around 6 weeks after the emergence of *An. coluzzii*, quickly vanishing with the drying-up of surface water. The population dynamics of both *An. gambiae s.s*. and *An. arabiensis* suggest immigration (using reliable wind systems) from southerly sources, where breeding sites are perennial. Based on these findings, minimal HFAs in *An. coluzzii* but elevated HFAs in both *An. gambiae s.s.* and *An. arabiensis*, were predicted to occur mostly during the wet season. Additionally, because long-distance migration in most insect species occurs before reproduction [2, 36, 58, 66], elevated HFAs in non-blood-fed females were expected, compared to gravid females. Unlike freshly blood-fed females which are burdened by the largest weight due to high water content of the bloodmeal, gravid females’ weight is intermediate and closer to the weight of the unfed female than to her weight when fully engorged [63]. Combined with the additional energy reserves that the bloodmeal offers, gravid females may be well suited to embark on long flights [45, 63]. There may be additional benefits to additional mass dependent on the flight modality while propelled by the wind such as gliding, soaring, which additional studies may uncover [67]. Finally, adding nutritional analysis with future tethered flight assays may shed light on the energetic content before and after the flight and possibly the allocation of nutritional reserves between reproduction and flight.

Overall, the results agree with the predictions based on the aerial sampling results, specifically regarding (1) elevated HFA during the wet season; (2) elevated HFAs among gravid mosquitoes; and (3) the presence of HFAs among all species, with highest flight aptitude in *An. coluzzii* and lowest flight aptitude in *An. arabiensis*. However, uncertainty remains, due to partial consistencies concerning the different flight aptitude indices, as well as coarse discrimination as a result of low statistical power among groups (Additional file 1: Table S1). For example, this uncertainty is reflected in the statistically non-significant differences in *An. coluzzii* between the early dry season (December–February) and the late dry season (March–April, Fig. 5), as well as the statistically non-significant difference between *An. gambiae* s.s. and the other two species (Fig. 6).

### Wing morphometry and flight aptitude

Morphological differences between wings of HFAs and LFAs can provide strong evidence in support of migrators classification while on the ground and reveal distinct developmental program(s) for long-distance migrants. The findings show that during the wet season, *An. coluzzii* HFAs had a larger wing area, attributable to an increase in both wing width and length, when compared with the LFAs. These results were not confounded by variation in body size between seasons as the analysis was confined to the wet season, when no seasonal change in wing length was detected (Additional file 1: Fig. S3, in agreement with previous results [63, 64]). The larger wings of HFAs may reflect isometric increases in all aspects of body size; alternatively, it may indicate an allometric change (e.g., an increase in wing area independent of body size). This is difficult to resolve with wild, mostly gravid mosquitoes due to the fact that variations in dry weight may confound bloodmeal size (and number) with body size. However, the allometric increase in wing width (over that expected by wing length) of *An. coluzzii* HFAs during the wet season further supports the validity of the classification and suggests that migrants undergo a distinct developmental plan prior to adult eclosion/emergence.

### Interpretation of flight behaviour

The prediction of higher flight activity during the wet season may sound counter-intuitive at first but fits with other empirical results and with theoretical expectations; No evidence to-date has shown high altitude flight in the early wet seasons (May–June). Huestis et al. [30] have shown that *An. coluzzii* and *An. gambiae s.s*. were collected in altitude from late July through November, similarly to flight aptitude results presented here. As discussed in Huestis et al. [30] the migration during the mid-wet season (July–September) probably follows the changing resources generated by the patchwork of precipitation that falls along the ITCZ as it sweeps through the Sahel northwards and then southwards. Unlike the early part of the season, in the later part of the wet season, i.e. October–November, the increase in elevated flight activity may represent individuals embarking on southerly, return flights before the dry season onset. Accordingly, results presented, both from altitude and the ground, support migration ‘within the Sahel’ and possibly emigration from the Sahel in October–November, when winds carrying insect southwards are more common. Additionally, these results indicate a similar capacity of these species for long-distance migration. The absence of evidence for high altitude migration during May–June may be due to the fact that during this period long-distance flight is suppressed, as conditions in Thierola are optimal, (i.e., minimal crowding, predation, competition) until local density increases (in late July when the ITCZ may well be some distance away), forming new optimal resources elsewhere. According to this hypothesis, elevated emigration will be detected in populations south of Thierola, maybe even south of Bamako, where the ITCZ (and the rains) arrives a month or so earlier.

In some species windborne migration appears to be a mandatory phase of the young adult [68], however, based on the low fraction of HFA (based on total flight), the members of the *An. gambiae* complex are considered here as an example of ‘partial migrators’ [38, 57], in which the majority of individuals in the population do not engage in long-distance migration, even during times when migration peaks. Moreover, after arrival, immigrants will exhibit LFA; therefore, these results may identify only emigrants prior to their journey. Since the migratory phase may last only 1–3 days, we would expect to have only 1–2 days, at most, to capture a migrant before they embark on their journey. Thus, a large sample size is required to represent migrants among the more numerous ‘appetitive flyers’, which adds noise to the data and limits the statistical power of detecting differences or trends.

To date, no information is available concerning mosquito flight behaviour at high altitudes. Even if tethered flight assays accurately identify long-distance migrants, the flight data generated in the assay is unlikely to mirror free flight behaviour of mosquitoes at altitude. For example, the total flight duration may greatly underestimate actual flight in altitude—simulations based on aerial sampling data [30] suggests night-long migratory flights in some cases. Likewise, flight-mill results may fail to exhibit flight patterns matching expectations based on migration due to technical, as well as biological reasons [31, 35, 37, 39]. Fair examples might include a lack of lift generation and a lack of tarsal contact, both of which may lead to unrealistically extended flight. On the other hand, the lack of sensory cues from either air movement, temperature and humidity gradients, or apparent ground movement, may curtail flights. In the present study, the flight aptitude assay relied on fixed-tethered mosquitoes placed in 50 mL Falcon tubes to partially isolate them from surrounding environmental cues. As a result, they may express intrinsically driven flight, as previously suggested in studies regarding locusts and moths [69, 70]. Finally, flight assays for migrant identification will be more informative when additional information is gathered on each mosquito to assess agreement with other aspects of the migration syndrome, pertaining to optimal locomotor drive in young, pre-reproductive adults, with metabolism switching between flight characteristics and ovary development [36, 54, 58, 65, 71, 72]. Examples of additional information might include combining data on nutritional reserves (typically elevated before migration) necessary to fuel extended flight, responses to host or oviposition site cues (typically inhibited prior and during migration) [73], levels of cuticular hydrocarbons (presumably elevated prior to migration to enhance desiccation tolerance) [64], and transcriptome analyses along with morphometrical analyses of size/shape of wings, thorax, and spiracles.

## Conclusions

- A new field-focused flight assay was developed and tested in order to identify long-distance migrators (LDM) among the *An. gambiae s.l.*
- A year-long experiment of flight aptitude of wild *An. gambiae s.l.* females from the Sahel revealed that flight activity exhibited a skewed distribution, with 10-29.7% identified as putative LDMs.
- Similar to findings by Huestis et al. [30], the flight aptitude results revealed a higher fraction of High Flight Activity (HFA) during the wet season compared with the dry, higher HFA in *An. coluzzii* compared with *An. arabiensis*, and a higher fraction of HFA in gravid females compared with unfed females. Additionally, evidence that *An. coluzzii* HFAs exhibit changes in wing size and shape was found, supporting that changes in the larval habitat (e.g., crowding) induce a specific developmental pattern yielding the HFA adult.
- Altogether, these results provide partial support for the utility of flight aptitude assays in identifying LDMs. Further studies should include a) optimization of the method to identify LDMs by integration of this assay with other assays that measure the “migratory syndrome” such as lipid deposits, withholding of blood-feeding, withholding of oviposition, and using the improved method for comparing geographically distinct populations for the fraction of LDMs.

## Supplementary information

**Additional file 1: Figure S1.** Flight sound file output (spectrogram view) in Audacity (a) and Raven Pro 1.5 software (b). Energy frequency (y-axis) and time of flight during the assay (x-axis) allowed extraction of flight durations during the assay. Panel a shows continuous flight bout of > 70 min (in white), followed by a > 40-min rest period (in red). Panel b (top) shows > 120-min long continuous flight (in black) ending in several shorter bouts, whereas the bottom panel shows multiple bouts spanning 2–20 s. **Figure S2.** Spearman and Pearson correlations between the key flight aptitude indices: total flight, longest flight and flight bouts. Upper row: scatter plots of flight aptitude indices pairs showing linear regression as trend lines (dark blue) across all data (N = 707 mosquitoes). Pearson and Spearman correlation coefficients are given with their significance level (*** and ns denotes P < 0.001 and P > 0.05, in Pearson (p) and Spearman (s) coefficients respectively). Lower row: Scatter plots as above, showing the relationships for HFA mosquitoes (red; n = 261) and LFA mosquitoes (blue; n = 446). HFAs were defined based on any one significant flight aptitude index (or more). **Figure S3.** Wing length in *Anopheles coluzzii* by time of year (season). Mean length represented as diamond symbols (◊). Medians as horizontal lines inside boxes. **Table S1.** Results summary table for the three flight aptitude indices: total flight, longest flight and flight bouts per test groups (top section). Shared high flight activity (HFA) frequency in the three flight aptitude indices (bottom section).

## Data Availability

The datasets during and/or analysed during the current study available from the corresponding author on reasonable request.

## Abbreviations

HFA(s): High flight activity (plural individuals exhibiting HFA)
LFA(s): Low flight activity (plural individuals exhibiting LFA)
RH: Relative humidity
*s.l.*: Sensu lato
*s.s.*: Sensu stricto
MAD: Median absolute deviation
MC: Monte Carlo (exact test)
CMH: Cochran–Mantel–Haenszel test
ANOVA: Analysis of variance
