# Supplementary material for: Quantifying flight aptitude variation in wild Anopheles gambiae in order to identify long-distance migrants
Source: Malar J. 2020 Jul 22;19:263. doi: 10.1186/s12936-020-03333-2 (PMC7374819; doi:10.1186/s12936-020-03333-2)
Supplement: Supplementary file 1 — Additional file 1: Figure S1. Flight sound file output (spectrogram view) in Audacity (a) and Raven Pro 1.5 software (b). Energy frequency (y-axis) and time of flight during the assay (x-axis) allowed extraction of flight durations during the assay. Panel a shows continuous flight bout of > 70 min (in white), followed by a > 40-min rest period (in red). Panel b (top) shows > 120-min long continuous flight (in black) ending in several shorter bouts, whereas the bottom panel shows multiple bouts spanning 2–20 s. Figure S2. Spearman and Pearson correlations between the key flight aptitude indices: total flight, longest flight and flight bouts. Upper row: scatter plots of flight aptitude indices pairs showing linear regression as trend lines (dark blue) across all data (N = 707 mosquitoes). Pearson and Spearman correlation coefficients are given with their significance level (*** and ns denotes P < 0.001 and P > 0.05, in Pearson (p) and Spearman (s) coefficients respectively). Lower row: Scatter plots as above, showing the relationships for HFA mosquitoes (red; n = 261) and LFA mosquitoes (blue; n = 446). HFAs were defined based on any one significant flight aptitude index (or more). Figure S3. Wing length in Anopheles coluzzii by time of year (season). Mean length represented as diamond symbols (◊). Medians as horizontal lines inside boxes. Table S1. Results summary table for the three flight aptitude indices: total flight, longest flight and flight bouts per test groups (top section). Shared high flight activity (HFA) frequency in the three flight aptitude indices (bottom section). [file 12936_2020_3333_MOESM1_ESM.docx]

**Quantifying flight aptitude variation in wild *A. gambiae* s.l. in order to identify long-distance migrants**

Roy Faiman^1*^, Alpha S. Yaro^2^, Moussa Diallo^2^, Adama Dao^2^, Samake Djibril^2^, Zana L. Sanogo^2^, Margery Sullivan^1^, Asha Krishna^1^, Benjamin J. Krajacich^1^ and Tovi Lehmann^1^

^1^ Laboratory of Malaria and Vector Research, National Institute of Allergies and Infectious Diseases, The National Institutes of Health. Rockville, MD, USA

^2^ Malaria Research and Training Center, Faculty of Medicine, Pharmacy and Odonto-Stomatology. Bamako, Mali

*Corresponding author. Email: roy.faiman@nih.gov

Supplementary information file

Flight Sound Extraction

Flight bouts were identified visually in spectrogram view (Fig. S1a) on Audacity (<https://www.audacityteam.org/>) or Raven Pro 1.4 software (http://ravensoundsoftware.com/raven-downloads/), and questionable flights were confirmed by listening for flight sound directly on the sound file.

Figure S1. Spectrogram view.


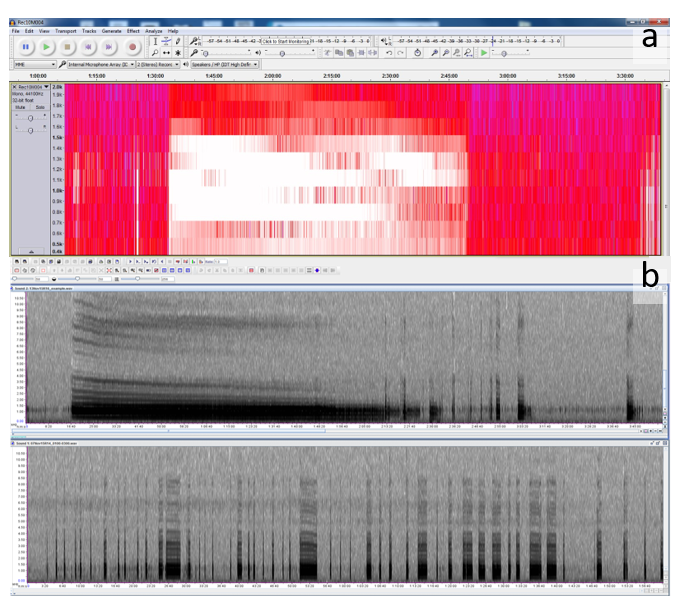


Figure S2. Flight Aptitude Indices relationships


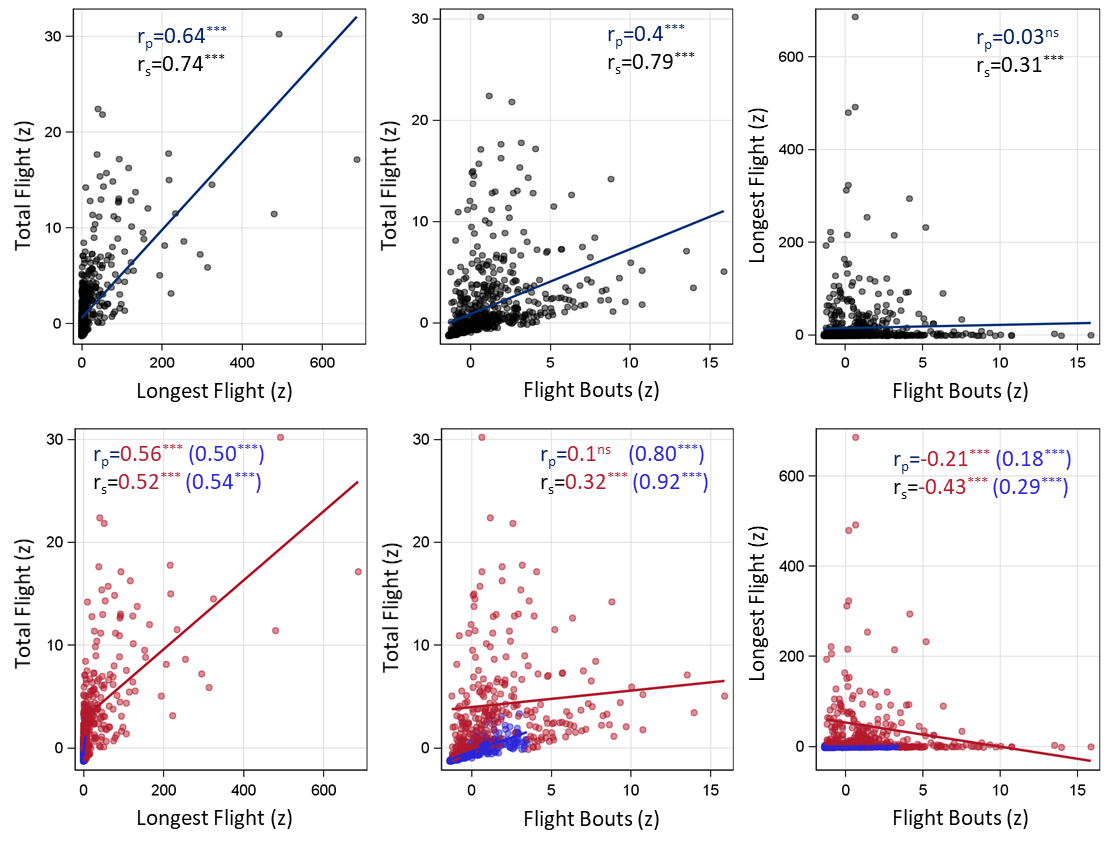


Wing size and Flight Aptitude

Wing length was putatively selected between the two points on the wing best representing its longest aspect (points 1 and 10). The wing width on the other hand, has no agreed upon set landmarks, and no two set points on the wing represent its width loyally. To overcome this, we selected several pairs of points on the wing, representing the proximal, medial and distal sections, and providing different distances. Assessing the degree of correlation between the three widths we eventually selected the mean of the three as the wing width.

Figure S3. Wing length in *Anopheles coluzzii* by time of year (season).


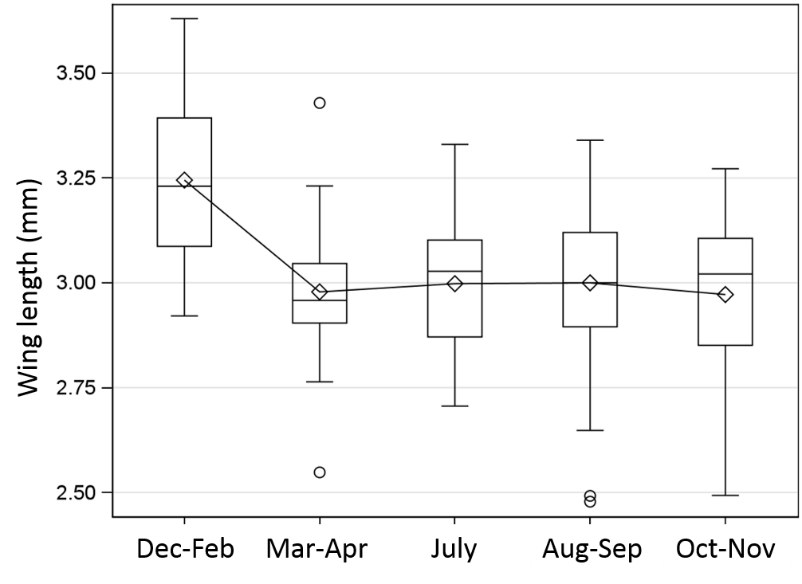


**Table S1.** Results summary table for the three flight aptitude indices: total flight, longest flight and flight bouts per test groups (top section). Shared high flight activity (HFA) frequency in the three flight aptitude indices (bottom section).

| **Group tests** | **Total Flight** | **Longest Flight** | **Flight Bouts** |
| --- | --- | --- | --- |
| Season ^a^ | * | ****** | reverse trend |
| Species ^b^ | * | similar trend | similar trend |
| Gonotrophic state ^c^ | similar trend | similar trend | * |
| Wing morphology (*A. coluzzii*, wet season) |  |  |  |
| Wing length | ****** | * | reverse trend |
| Wing width | ****** | * | reverse trend |
| Allometry | * | reverse trend | reverse trend |
| HFA Shared Frequency (%) ^d^ |  |  |  |
| Total Flight | 100 | - | - |
| Longest Flight | 90.3 | 100 | - |
| Flight Bouts | 23.9 | 10.5 | 100 |
| HFA Overall Frequency (%) ^e^ | 16.0 | 29.7 | 10.0 |
| * significance of P<0.05, ** significance of P<0.001 ^a^Oct-Nov > Aug-Sep = Jul = Dec-Feb > Mar-Apr ^b^A. coluzzii > A. gambiae > A. arabiensis ^c^Gravid > Unfed ^d^ Frequency of HFA mosquitoes in two FA indices ^e^ Total frequency of HFAs per FA index | | | |
